# BioMart – biological queries made easy

**DOI:** 10.1186/1471-2164-10-22

**Published:** 2009-01-14

**Authors:** Damian Smedley, Syed Haider, Benoit Ballester, Richard Holland, Darin London, Gudmundur Thorisson, Arek Kasprzyk

**Affiliations:** 1 European Bioinformatics Institute, Wellcome Trust Genome Campus, Hinxton, Cambridge, CB10 1SD, UK; 2 Institute for Genome Sciences & Policy (IGSP), Duke University CIEMAS, 101 Science Drive, DUMC Box 3382, Durham, NC 27708, USA; 3 Department of Genetics, University of Leicester, University Road, Leicester, LE1 7RH, UK; 4 Ontario Institute for Cancer Research, MaRS Centre, South Tower, 101 College Street, Suite 800 Toronto, Ontario, M5G 0A3, Canada

## Abstract

**Background:**

Biologists need to perform complex queries, often across a variety of databases. Typically, each data resource provides an advanced query interface, each of which must be learnt by the biologist before they can begin to query them. Frequently, more than one data source is required and for high-throughput analysis, cutting and pasting results between websites is certainly very time consuming. Therefore, many groups rely on local bioinformatics support to process queries by accessing the resource's programmatic interfaces if they exist. This is not an efficient solution in terms of cost and time. Instead, it would be better if the biologist only had to learn one generic interface. BioMart provides such a solution.

**Results:**

BioMart enables scientists to perform advanced querying of biological data sources through a single web interface. The power of the system comes from integrated querying of data sources regardless of their geographical locations. Once these queries have been defined, they may be automated with its "scripting at the click of a button" functionality. BioMart's capabilities are extended by integration with several widely used software packages such as BioConductor, DAS, Galaxy, Cytoscape, Taverna. In this paper, we describe all aspects of BioMart from a user's perspective and demonstrate how it can be used to solve real biological use cases such as SNP selection for candidate gene screening or annotation of microarray results.

**Conclusion:**

BioMart is an easy to use, generic and scalable system and therefore, has become an integral part of large data resources including Ensembl, UniProt, HapMap, Wormbase, Gramene, Dictybase, PRIDE, MSD and Reactome. BioMart is freely accessible to use at .

## Background

In this post-genomics era, data of increasing volume and complexity is being deposited into databases around the world. Biologists need to ask complex queries of this data to test and drive their research hypotheses. Typically, each data source provides an advanced query interface on their website to satisfy this requirement. However, each site has its own solution and subsequently, the user has a learning curve before they can start interacting with the data. A further problem the researcher has is that they often need to query more than one data source, necessitating mastering more than one interface and having to cut and paste results between the sites. If the analysis involves high-throughput data, this approach is not usually scalable. To overcome this problem, many groups rely on bioinformaticians who can generate scripts to interact with the varying programmatic interfaces of the different data sources. They also often have to learn a number of different web services or application programmatic interfaces (APIs) for each resource. A preferable solution would be to have generic software that a biologist can use on top of any data source. BioMart[1] is such a solution.

BioMart is an open source data management system that comes with a range of query interfaces that allow users to group and refine data based upon many different criteria. In addition, the software features a built-in query optimiser for fast data retrieval. A BioMart installation can provide domain-specific querying of a single data source or function as a one-stop shop (web portal) to a wide range of BioMarts as our central portal [2] does. All BioMart websites have the same look and feel (only varying in colour scheme and branding), which has obvious advantages to users moving between different resources. However, the power of the system comes from integrated querying of the different BioMarts. If any datasets share common identifiers (such as Ensembl gene IDs or Uniprot IDs) or even mappings to a common genome assembly, these can be used to link BioMarts together in integrated queries. Additionally, these datasets do not have to be located on the same server or even at the same geographical location. This distributed solution has many advantages; not least of which is the fact that each site can utilise their own domain expertise to deploy their BioMart.

BioMart also has the advantage of being integrated with external software packages such as BioConductor [3], the Distributed Annotation System (DAS) [4], Galaxy [5], Cytoscape [6], Taverna [7]. This enables users to perform integrated queries with non-BioMart data sources as well as detailed analysis of the results. BioMart is also part of the GMOD (Generic Model Organism Database) [8] suite of tools for building a model organism site.

Originally developed for the Ensembl genome browser [9] as the EnsMart data warehouse [10], BioMart has now become a fully generic data integration solution. Although applicable to any type of data, BioMart is particularly suited for advanced searching of the complex descriptive data typically found in biological datasets. Numerous BioMarts have now been installed by external groups, in large part because of its automated deployment tools and cross platform compatibility. These include model organism databases such as Gramene [11], Dictybase [12], Wormbase [13] and RGD (Rat Genome Database) [14] as well as HapMap variation [15], pancreatic expression database [16], Reactome pathways [17] and PRIDE proteomic [18] databases (see Table 1 for the full list). A wide variety of analyses and tasks are possible from the publicly available BioMarts, ranging from SNP (single nucleotide polymorphism) selection for candidate gene screening, microarray annotation, cross-species analysis, through to recovery of disease links, sequence variations and expression patterns.

**Table 1:** Description of all publicly accessible BioMarts to date

**Name of BioMart**	**Description of contents**	**Location of BioMart**
Ensembl Genes	Automated annotation of over 40 eukaryotic genomes	EMBL-EBI, UK
Ensembl Homology	Ensembl Compara orthologues and paralogues	EMBL-EBI, UK
Ensembl Variation	Ensembl Variation data from dbSNP and other sources	EMBL-EBI, UK
Ensembl Genomic Features	Ensembl Markers, clones and contigs data	EMBL-EBI, UK
Vega	Manually curated human, mouse and zebrafish genes	EMBL-EBI, UK
HTGT	High throughput gene targeting/trapping to produce mouse knock-outs	Sanger, UK
Gramene	Comparative Grass Genomics	CSHL, USA
Reactome	Curated database of biological pathways	CSHL, USA
Wormbase	*C. elegans *and *C. briggsae *genome database	CSHL, USA
Dictybase	Dictyostelium discoideum genome database	Northwestern University, USA
RGD	Rat model organism database	Medical College of Wisconsin, USA
PRIDE	Proteomic data repository	EMBL-EBI, UK
EURATMart	Rat tissue expression compendium	EMBL-EBI, UK
MSD	Protein structures	EMBL-EBI, UK
Uniprot	Protein sequence and function repository	EMBL-EBI, UK
Pancreatic Expression Database	Pancreatic cancer expression database	Barts & The London School of Medicine, UK
PepSeeker	Peptide mass spectrometer data for proteomics	University of Manchester, UK
ArrayExpress	Microarray data repository	EMBL-EBI, UK
GermOnLine	Cross species knowledgebase of genes relevant for sexual reproduction	Biozentrum/SIB, Switzerland
DroSpeGe	Annotation of 12 Drosophila genomes	Indiana Univeristy, USA
HapMap	Catalogue of common human variations in a range of populations	CSHL, USA
VectorBase	Invertebrate vectors of human pathogens	University of Notre Dame, USA
Paramecium DB	Paramecium tetraurelia model organism database	CNRS, France
Eurexpress	Mouse *in situ *expression data	MRC Edinburgh, UK
Europhenome	Mouse phenotype data from high throughput standardized screens	MRC Harwell, UK

The range of interfaces is designed with both biologists and bioinformaticians in mind. The simplest way of querying BioMart is via the web interface called MartView (either on our central portal [2] or follow the links on our main page [1] to the individual sites). Programmatic access is available via a Perl API or BioMart's web services (MartServices). An important and novel feature of BioMart is that it offers "scripting at the click of a button". A user can generate an API or MartServices script by building up a query on the MartView website followed by a simple click of a button. All the interfaces allow the user to build up biological queries by first selecting **dataset(s) **of interest, then the data to view and/or save (**attributes**), some optional restrictions (**filters) **on the query and finally the **format **for the data.

## Implementation

Here we will describe a top-level view of the BioMart system as the focus of this paper is on practical use of BioMart rather than implementation and deployment. Further documentation on these aspects can be found on the BioMart website . BioMart is designed around a simple, three tier architecture:

(i) The first tier consists of one or more relational databases. Each database may consist of one or more "marts", which are schema compliant with BioMart definitions. Each "mart" may hold a number of different datasets. The BioMart data model is a denormalised, query optimised schema and can be deployed using Oracle, MySQL or Postgres relational database management systems. Each dataset uses a reversed star model [10] where data mapping 1:1 with the central object being modelled is stored in a main table. Data mapping 1:n is stored in one or more satellite, dimension tables. Two tools are provided to build and configure the mart databases in the first tier:

• MartBuilder, to construct SQL statements that will transform your schema into a mart.

• MartEditor, to configure the finished mart for use with the rest of the system. This produces a dataset configuration XML (Extensible markup language) that is stored in metadata tables within the actual mart database.

(ii) The second tier is the Perl API (distributed in the biomart-perl package) which interacts with both the dataset configuration and the mart databases.

(iii) The third tier consists of the query interfaces which utilise the API to present the possible BioMart queries and results:

• MartView, a web browser interface.

• MartService, a web services interface.

• MartURLAccess, a URL based access to MartView.

## Results

### (i) BioMart website

In this section we describe how to use the MartView web interface with an example, followed by a few more biologically relevant queries that users may perform with the currently available mart interfaces. For the first example, we show how to retrieve "the Ensembl mouse genes and genomic locations in the first 10 Mbp of chromosome 1 region that have already been targeted as part of the International Mouse Mutagenesis Consortium ".

**Figure 1:**
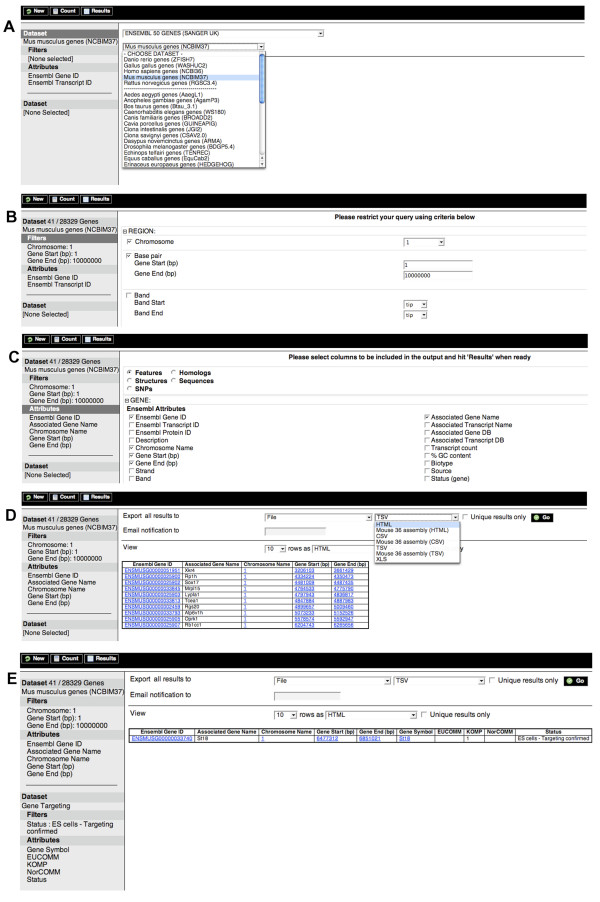
**BioMart query showing that the St18 gene is the only mouse gene in the first 10 Mb of chromosome 1 that is annotated as "targeting complete" by the International Mouse Mutagenesis Consortium**. This involves: (A) selecting the Ensembl *Mus musculus *genes dataset, (B) setting the filters, (C) setting the attributes, (D) viewing the results, and (E) adding in the Gene targeting dataset to obtain just the gene that has reached the "targeting complete" status.

MartView is reached by clicking the link from the BioMart site [1]. The query is started by selecting the *ENSEMBL GENES *database and the *Mus musculus genes *dataset (Figure 1A) hosted at the EBI, UK. Next filters to refine the query are set by clicking on the Filters bar in the left hand pane, expanding the *REGION *section in the main right hand pane and setting the *Chromosome, Gene Start *and *Gene End *filters to 1, 1 and 10,000,000 respectively (Figure 1B). To find out how many genes would be returned at this point, use the Count button in the menu bar. Next, the user should choose the data fields they want to view or download in a similar manner by clicking on the Attributes bar in the left hand pane and choosing *Ensembl Gene ID, Associated Gene name, Chromosome Name, Gene Start (bp), Gene End (bp) *in the *GENE *section (Figure 1C). Note that attributes and filters can be selected in any order. The left pane shows the summary of datasets, attributes and filters chosen. They will appear in the order of selection, and this same order will be used to organise the results later. Finally, the user needs to click on the Results button in the menu bar to get a preview of the results (Figure 1D). In this panel the number of rows to preview can be changed along with the format to preview them in e.g. hypertext markup language (HTML), Excel (XLS), FASTA, tab-separated values (TSV), comma-separated values (CSV), or gene structure format (GFF). From this panel the user may also export all the results to a file. Time-out settings at particular sites can cause problems, so for these cases there is a 'notify by email' option where the results are generated and stored on the server-side. When the results are ready, the user is sent an email containing a link to download the results. The 'Unique results only' option is useful for removing redundant rows in the output: for example if a user selected Ensembl Gene ID and another attribute mapped at the transcript level, this could happen.

The user now has the genomic locations of all the mouse genes in the first 10 Mbp of chromosome 1 but not whether the genes have been targeted yet in the mouse knockout project. To finish this example, a second dataset needs to be added to the query, this time retrieving mouse knockout data from the BioMart located at the Sanger Institute, UK. The second (lower) Dataset bar on the left hand pane has to be clicked and the *Gene targeting *dataset from the *HIGH THROUGHPUT GENE TARGETING AND TRAPPING *database selected. The *status *filter is then set to 'ES cells – Targeting confirmed' and the attributes set to *Gene symbol*, *EUCOMM, KOMP, NorCOMM *(component projects of the International Mouse Mutagenesis Consortium) and *status*. This time, when the results button is clicked, a single mouse gene (St18) in the chromosome 1 region is shown to be have reached the "targeting confirmed stage" and has been assigned to the KOMP project (Figure 1E).

Another common use of BioMart is the analysis of the genes up-regulated in a particular microarray experiment. For example, a user might retrieve "1 kb of upstream sequences from a cluster of human genes identified by an expression profile experiment on an Affymetrix Genechip U95Av2".

**Figure 2:**
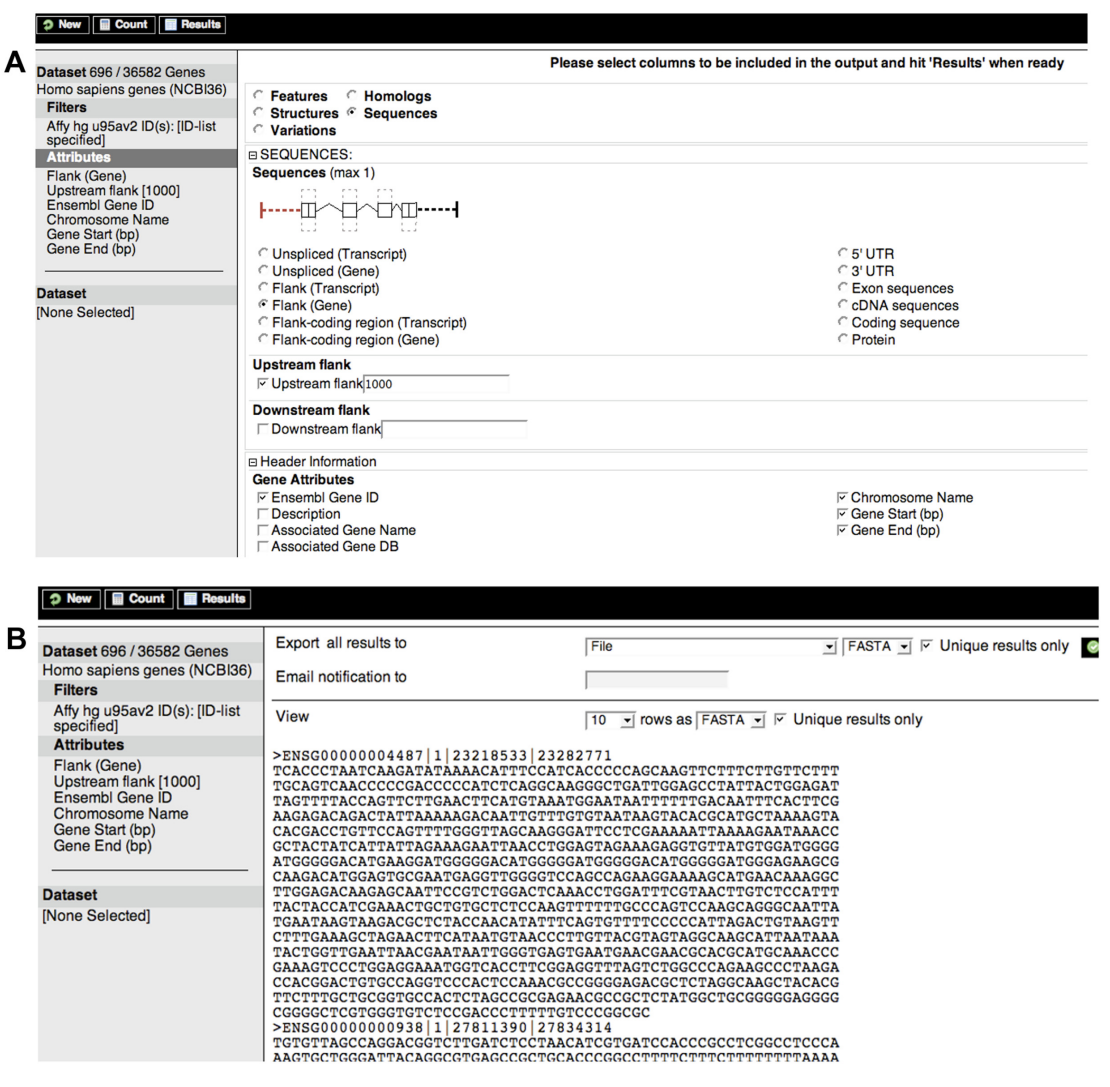
**(A) Sequence output options and (B) FASTA output for all the genes found to be up-regulated in a microarray experiment using the Affymetrix HG-U95Av2 probeset**. Here 1000 bp upstream of the first exon have been chosen along with the Ensembl Gene Id and the chromosomal position of the gene for the FASTA header.

This query is started with the New button on the menu bar to take the user to a new query page. The *Homo sapiens genes *dataset is selected, and filters selected by clicking on the Filter bar again but this time the *ID list limit *filter in the *GENE *section is chosen. Choosing the *Affy hg u95av2 ID(s) *option allows the user to upload a file of experimentally relevant Affymetrix probeset IDs from this Genechip using the file Browse button or to enter IDs by cutting and pasting into the text box (we include some example IDs in Additional file 1). The various sequence options can be seen by clicking on Sequences at the top of the page in the attributes section (Figure 2A). These include cDNA (complementary DNA), peptides, coding regions, UTRs (untranslated regions), and exons with additional upstream and downstream flanking regions. In order to identify upstream regulatory features in subsequent analysis, the user would select 1000 bp of upstream flank sequence for each gene (Figure 2B). Note that a variety of attributes can be selected for the FASTA header line of the sequence file.

BioMart may also be used for annotating experiments or for mapping identifiers to genes and vice a versa. For instance, instead of exporting sequence in the above example, the user could have chosen gene identifiers and names mapping to the uploaded microarray probe IDs. Ensembl contains a wide range of external identifiers for its genes making detailed annotation possible e.g. GO (Gene Ontology), EMBL (European Molecular Biology Laboratory)/Genbank, UniProt (Universal Protein Resource), UniGene, Pfam (Protein Family), PDB (Protein Data Bank) and RefSeq identifiers as well as official names from the naming committees of each species, such as the HGNC (HUGO Gene Nomenclature Committee) and MGI (Mouse Genome Informatics) symbols.

**Figure 3:**
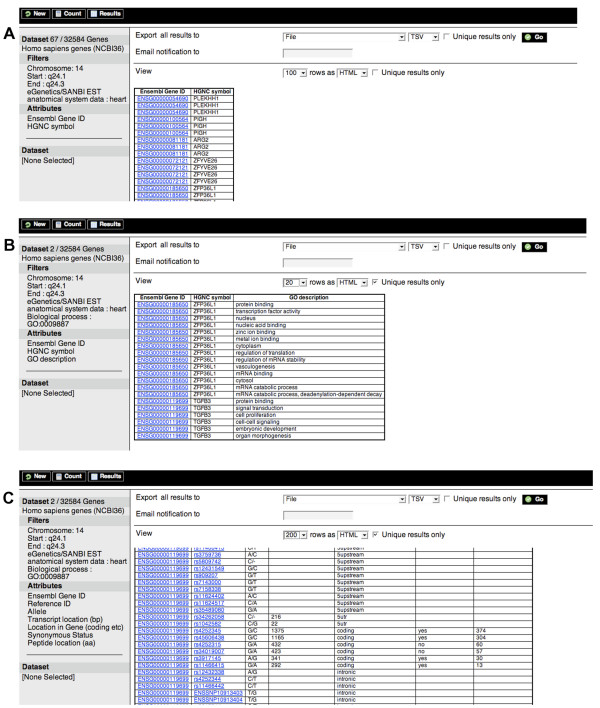
**Candidate gene identification using BioMart**. (A) The Arrhythmogenic right ventricular dysplasia (ARVD) gene was mapped to 14q24. BioMart identifies 172 genes in this region, which may be narrowed down to 67 with expression in the heart. (B) This may be further refined to the two candidate genes, ZFP36L1 and TGFB3, by looking for genes involved in organ morphogenesis, according to GO, as this condition is known to result in widespread structural abnormalities. The latter gene is now known to be the one involved in this disorder. (C) BioMart may also be used to extract SNPs for the identified genes including their location in the gene, whether they are upstream, downstream, intronic or coding and for the latter whether they result in an amino acid substitution.

Another typical use case for BioMart is in the identification of candidate genes for disease association. For example, a locus for Arrhythmogenic right ventricular dysplasia was originally mapped to 14q24 [19]. A list of 172 genes may be identified in this region, with 67 having expression in heart as assessed from EST (expressed sequence tag) derived data (Figure 3A). Exporting the GO description data for these candidate genes (Figure 3B) immediately reveals two potential candidates with a role in organ morphogenesis which is affected in this disorder: ZFP36L1 and TGFB3. TGFB3 was eventually shown to be mutated in the affected families [20]. BioMart enabled this complex query to be performed very quickly and easily. Following the identification of candidate disease associated genes, researchers often screen for SNPs showing an association with the disease. BioMart provides a quick way of identifying suitable SNPs to screen. For each of the candidate genes, the user can export a list of the SNP identifiers mapped within that gene, and SNP attributes such as their location in the transcript and coding sequence, and whether they are non-synonymous (together with the associated amino acid change) (Figure 3C).

Recently, many other groups have applied the BioMart technology to help their scientists answer complex queries such as those shown above. For example, the CASIMIR (Coordination and Sustainability of International Mouse Informatics Resources) consortium has created a mouse portal prototype [21].

### (ii) Scripting at the click of a button

#### (a) Perl API

The Perl API (for download and install instructions see [22]) is self-explanatory with the help of an example. The best way to learn and, indeed, generate the API scripts is to use the Perl button in the top pane of any MartView site after a manual query has been defined. The script below extracts the mouse and human Ensembl Gene ID and genomic positions for all human genes on chromosome 1 that have a mouse orthologue on chromosome 2. Just like the website, generating a query involves setting a dataset, adding filters and attributes and selecting an output format. The user can even get a count of the results just like the website.

use strict;

use BioMart::Initializer;

use BioMart::Query;

use BioMart::QueryRunner;

my $confFile = "PATH TO YOUR REGISTRY FILE UNDER biomart-perl/conf/"

my $initializer = BioMart::Initializer-> new('registryFile'=>$confFile, 'action'=>'cached');

my $registry = $initializer->getRegistry;

my $query = BioMart::Query-> new('registry'=>$registry,'virtualSchemaName'=>'default');

$query->setDataset("hsapiens_gene_ensembl");

$query->addFilter("chromosome_name", ["1"]);

$query->addAttribute("ensembl_gene_id");

$query->addAttribute("chromosome_name");

$query->addAttribute("start_position");

$query->addAttribute("end_position");

$query->setDataset("mmusculus_gene_ensembl");

$query->addFilter("chromosome_name", ["2"]);

$query->addAttribute("ensembl_gene_id");

$query->addAttribute("chromosome_name");

$query->addAttribute("start_position");

$query->addAttribute("end_position");

$query->formatter("TSV");

my $query_runner = BioMart::QueryRunner->new();

# to obtain count

# $query->count(1);

# $query_runner->execute($query);

# print $query_runner->getCount();

# to obtain unique rows only

# $query_runner->uniqueRowsOnly(1);

$query_runner->execute($query);

$query_runner->printHeader();

$query_runner->printResults();

$query_runner->printFooter();

#### (b) MartServices

MartServices, BioMart's RESTful type web services, is available as part of the MartView web application and, as with all the BioMart interfaces, designed to be as simple to use as possible. It is available from the same location as MartView, i.e. if the user accesses MartView using  then they would access the MartServices using . Both overview information (metadata) about the services and queries are submittable (for details on the metadata services see the documentation on the main site[1]).

Like the Perl API, the query XML for MartServices is self-explanatory and again the best way to learn and, indeed, generate it is to use the XML button in the top pane of any MartView site to produce a representation of the currently configured query. The XML to recreate the Perl API example above (extracting the mouse and human Ensembl Gene ID and genomic positions for all human genes on chromosome 1 that have a mouse orthologue on chromosome 2) is shown below:

<?xml version="1.0" encoding="UTF-8"?>

<!DOCTYPE Query>

<Query virtualSchemaName = "default" formatter = "TSV" header = "0" uniqueRows = "0" count = "" datasetConfigVersion = "0.6" >

      <Dataset name = "hsapiens_gene_ensembl" interface = "default" >

            <Filter name = "chromosome_name" value = "1" />

            <Attribute name = "ensembl_gene_id" />

            <Attribute name = "chromosome_name" />

            <Attribute name = "start_position" />

            <Attribute name = "end_position" />

      </Dataset>

      <Dataset name = "mmusculus_gene_ensembl" interface = "default" >

            <Filter name = "chromosome_name" value = "2" />

            <Attribute name = "ensembl_gene_id" />

            <Attribute name = "chromosome_name" />

            <Attribute name = "start_position" />

            <Attribute name = "end_position" />

      </Dataset>

</Query>

Again, note how the query is formed by adding Datasets within a Query Tag and filters and attributes within the Datasets. As for the Perl API the output format can be changed with a formatter setting, counts can be performed by setting count="1" and unique rows by setting uniqueRows="1" on the Query. To submit a query to MartServices, this XML has to be posted to  by appending a query parameter. For example using wget: wget – O results.txt '' replacing MY_XML with the XML obtained above.

#### (c) MartView URL/XML requests

The MartView web interface can be pre-populated with an existing query using URL/XML requests. This can be achieved by sending the XML query as described in the previous section to the following URL



Equivalently, an XML free representation of the same query can be sent to the following URL for similar results



<XML_REQUEST> can be replaced with the XML query as described above for MartServices and <URL_REQUEST> represents the same query in a URL format. As for the Perl API and MartService interfaces, construction of the URL request is best handled by building the query using the MartView website and then using the URL button in the top pane of MartView. The URL/XML request feature is obviously useful for bookmarking favourite queries as well as for constructing canned queries when linking from an external site directly to MartView.

### (iii) BioMart integration with external software

A number of external software packages have incorporated BioMart querying capabilities into their systems. Generally, this has been carried out to improve their software by importing data through BioMart into their system for: (i) further analysis using the existing services they provide (Galaxy, BioConductor, Taverna) or (ii) to add further annotation to their results (Cytoscape). This integration has been made possible through MartServices. All the requests generated by these external packages run against the BioMart central portal. Using BioMart through these external packages expands the usefulness of both BioMart and these external tools. Therefore, brief descriptions and examples of this integrated usage are presented below. BioMart has also been improved by the incorporation of external software technologies. BioMart can be easily configured to become a DAS annotation server for viewing of data through various DAS clients.

#### (a) Galaxy

Galaxy [5,23] integrates genomic sequences, alignments and functional annotation through an interactive and simple web portal, so no installation is required. The system allows users to gather data using resources like BioMart or the UCSC (University of California Santa Cruz) Table Browser. The user can then manipulate the data in a variety of ways, such as simple intersections (e.g. selecting the genes common to two BioMart result sets), unions, and subtractions, or more complex analysis using tools such as those from the EMBOSS (European Molecular Biology Open Software Suite) package [24]. An example of this integrated analysis is shown in Additional file 2.

#### (b) BioConductor

BioConductor [3] is open source software for the analysis of genomic data. BioConductor is based on the R programming language that is especially suited for statistical analysis. Comprehensive instructions on how to install R and BioConductor are provided on their site [25]. The *biomaRt *package provides an API to query BioMart databases for use within BioConductor.

*biomaRt *mimics the functionality of the Perl API, allowing retrieval of any of the information that the other BioMart interfaces allow. A second set of functions is tailored towards Ensembl and include commonly used queries in microarray data analysis. Using *biomaRt*, a user can for example annotate the features on an array with official gene names, GO identifiers/descriptions and OMIM (Online Mendelian Inheritance in Man) terms retrieved via identifiers, such as Affymetrix, Locuslink, RefSeq or EntrezGene IDs. The package also provides homology mappings between these identifiers across all the species present in Ensembl.

The first stage in using biomaRt is to load the library and choose a mart to use:

library(biomaRt)

listMarts()

The results of this command are shown in Table 2

**Table 2:** Output from the listMarts command of the BiomaRt library

	**name**	**version**
**1**	**ensembl**	**ENSEMBL 49 GENES (SANGER)**
**2**	**compara_mart_homology_49**	**ENSEMBL 49 HOMOLOGY (SANGER)**
**3**	**compara_mart_pairwise_ga_49**	**ENSEMBL 49 PAIRWISE ALIGNMENTS**
**4**	**compara_mart_multiple_ga_49**	**ENSEMBL 49 MULTIPLE ALIGNMENTS**
**5**	**Snp**	**ENSEMBL 49 VARIATION (SANGER)**
**...**		

Next a dataset is chosen:

ensembl = useMart("ensembl")

listDatasets(ensembl)

The results of this command are shown in Table 3

**Table 3:** Output from the listDatasets command of the BiomaRt library

	**dataset**	**version**
**1**	**oanatinus_gene_ensembl**	**OANA5**
**2**	**gaculeatus_gene_ensembl**	**BROADS1**
**...**		

To set the dataset to be queried, the useMart function is used:

human = useMart("ensembl", dataset="hsapiens_gene_ensembl")

The query is constructed with the getBM function. For example, the following will return the Ensembl Gene ID and genomic position for genes up-regulated in an Affymetric HG U133 Plus 2 experiment:

getBM(attributes=c("ensembl_gene_id","chromosome_name","start_position","end_position"),filter="affy_hg_u133_plus_2",values=c('215984_s_at', '203174_s_at', '215984_s_at'), mart=human)

The results of this command are shown in Table 4

**Table 4:** Output from the getBM command of the BiomaRt library

	**ensembl_gene_id**	**chromosome_name**	**start_position**	**end_position**
**1**	**ENSG00000026036**	**20**	**61759607**	**61800495**
**2**	**ENSG00000101246**	**20**	**61801253**	**61809809**

#### (c) Cytoscape

Cytoscape [6] is open source software for the visualisation of molecular interaction networks and their integration with other biological data such as gene expression profiles. Cytoscape uses both web services and BioMart MartServices to retrieve this extra annotation (see Additional file 3).

#### (d) Taverna workbench

The Taverna workbench [7,26], another open source software package which integrates BioMart, allows interoperation between local and remote analysis tools and databases by providing an environment for the design and execution of workflow experiments. Taverna is able to utilise BioMart, web services and BioMoby [27] services allowing its users to combine over 3000 different resources and analysis tools, providing a flexible and extensible platform for bioinformatics research. Overall, Taverna allows bioinformaticians to build automated protocols that access each data source and integrate the collected results into a suitable form for biologists to explore (see Additional file 4).

#### (e) Distributed annotation system

Any BioMart server can be easily configured to act as a DAS annotation server [4] so that any DAS client such as GBrowse or the Ensembl genome browser can display the data stored in BioMart. DAS offers a simple system for data federation where a DAS client may be used to view the data from several sources in a single, usually graphical, interface. For example, genes stored in a BioMart dataset, such as those affected in a mouse strain stored in the European Mouse Mutant Archive (EMMA) repository may be displayed as tracks in Ensembl contigView along with the usual gene tracks (see Additional file 5).

A list of the sources available from our central portal is available [28]. This server currently returns annotation across a 'segment'. Possible 'segment' values could be a feature identifier or a genomic region defined by chromosome:start,end whereby start and end are optional. For example, an ensembl Homo sapiens DAS data source called 'default__hsapiens_gene_ensembl__ensembl_das_gene' can be accessed as:







## Discussion

An important feature we intend to implement in the near future is secure data access. This is vital where certain data is sensitive and adding this feature will make BioMart an even more attractive solution for organisations looking for controlled access. Adoption of BioMart will then allow secure and simple browsing of their private data, as well as the power of integrated querying of this data with the available public BioMarts.

We will also focus on new interfaces that further simplify multiple dataset querying. In these new interfaces, once dataset(s) are chosen, all the attributes and filters will be presented to the user as if from a single data source, even if they originate from distributed BioMarts. These interfaces will also address the needs of users who require a simpler, more limited, query tool. In addition, we will expand the options available for viewing, analysing and saving results. For example, we will offer gene reports containing all the information we have on a gene in a nicely formatted web page. We will also offer graphical display of results such as locations on a karyogram or a bar chart display of distributions. Furthermore, statistical analysis of results will be provided e.g. whether particular GO terms are enriched in the result set.

## Conclusion

In this paper, we have demonstrated that BioMart provides a range of simple, but powerful, interfaces for querying biological data. These may be used for many important research applications such as the data mining of large genomic resources, identification of candidate disease associated genes and variations within them, and the annotation of genome-wide experiments such as microarray studies. BioMart's architecture allows integrated querying of resources at different locations. As the number of publicly available BioMarts increases, users will be able to ask ever more complex queries. The presence of a services layer (MartService) and Perl API provides easy programmatic access for more technical users to script against BioMart and to integrate our software into their own systems. However, we hope the simplicity of generating the MartService and Perl API queries via the website will encourage the novice user to use these interfaces where appropriate. The integration of BioMart with external software adds a further dimension to its usefulness as a research tool. BioMart is also part of GMOD. This project aims to provide a free set of software for creating and administering a model organism database, including genome visualisation, annotation and literature curation. Tools currently exist to produce a BioMart database from the GFF3 gene structure files commonly used by GMOD and further integration of BioMart and other components of GMOD is planned in the near future.

We hope that this description of BioMart, and the direction the system is heading, will have encouraged users and data deployers to explore BioMart for their own biological query requirements.

## Availability and requirements

Text Project name: BioMart

Project home page: 

Operating system(s): Any. Local deployment of BioMart requires the Java Virtual Machine 1.3,1.4 or 1.5, Perl 5.6.0 or higher and either Apache 1.3,1.4 or Apache 2.0 or higher are required

Programming language: Java and Perl

License: LGPL

Any restrictions to use by non-academics: None.

## Authors' contributions

DS, SH, BB, RH, DL, GT have all contributed to the development of AK's vision of an easy-to-use system for advanced querying of biological data. AK continues to drive the design and development of BioMart at his new position at the Ontario Institute for Cancer Research with contributions from SH. The manuscript was drafted by DS, SH, BB with all authors contributing revisions and with DS coordinating the effort. All authors read and approved the final version of the manuscript.

## Supplementary Material

Additional file 1 **Sample Affymetrix Probe IDs.** File of Affymetrix HG U95AV2 IDs for the BioMart example given in the paper. Click here for file

Additional file 2 **Use of BioMart within the Galaxy system.** BioMart embedded in the Galaxy framework is used to retrieve the peptide sequence for the mouse Bambi gene (A). The peptide sequence is saved on the Galaxy server and then transmembrane domains identified in it by running tmap analysis (part of the EMBOSS package) also from within Galaxy (B). The downloaded results file shows two potential transmembrane segments (C). Click here for file

Additional file 3 **Cytoscape platform used to visualise a yeast protein interaction network.** Annotation of the selected nodes in yellow is shown in the bottom pane and uses MartServices on our central portal to retrieve the GO annotation for each node. Click here for file

Additional file 4 **Taverna workflow demonstrating BioMart and web services interaction.** Ensembl Gene IDs and EMBL IDs for a given set of genes (results of an Affymetrix microarray experiment) are recovered. The left hand panel shows a graphical depiction of the workflow in which the EMBL IDs are converted to KEGG IDs and then HTML links to marked up pathways using KEGG web services. The upper right panel shows the tabular results of the workflow with Ensembl Gene IDs mapped to KEGG pathway URLs. The bottom right panel shows one of these links with the mapped gene marked in red in the pathway. Click here for file

Additional file 5 **BioMart as a DAS server.** Ensembl ContigView display showing a EMMA mouse strain archive track in blue (A). The data is served using the DAS protocol from a BioMart server in an external location to the rest of the Ensembl data. Ensembl GeneView showing Pancreatic Expression Database annotation (B). This annotation comes from a geneDAS source served by the BioMart server. Click here for file
