# Supplementary material for: BioMart – biological queries made easy
Source: BMC Genomics. 2009 Jan 14;10:22. doi: 10.1186/1471-2164-10-22 (PMC2649164; doi:10.1186/1471-2164-10-22)
Supplement: Additional file 2 — Use of BioMart within the Galaxy system. BioMart embedded in the Galaxy framework is used to retrieve the peptide sequence for the mouse Bambi gene (A). The peptide sequence is saved on the Galaxy server and then transmembrane domains identified in it by running tmap analysis (part of the EMBOSS package) also from within Galaxy (B). The downloaded results file shows two potential transmembrane segments (C). [file 1471-2164-10-22-S2.doc]

| **A** | 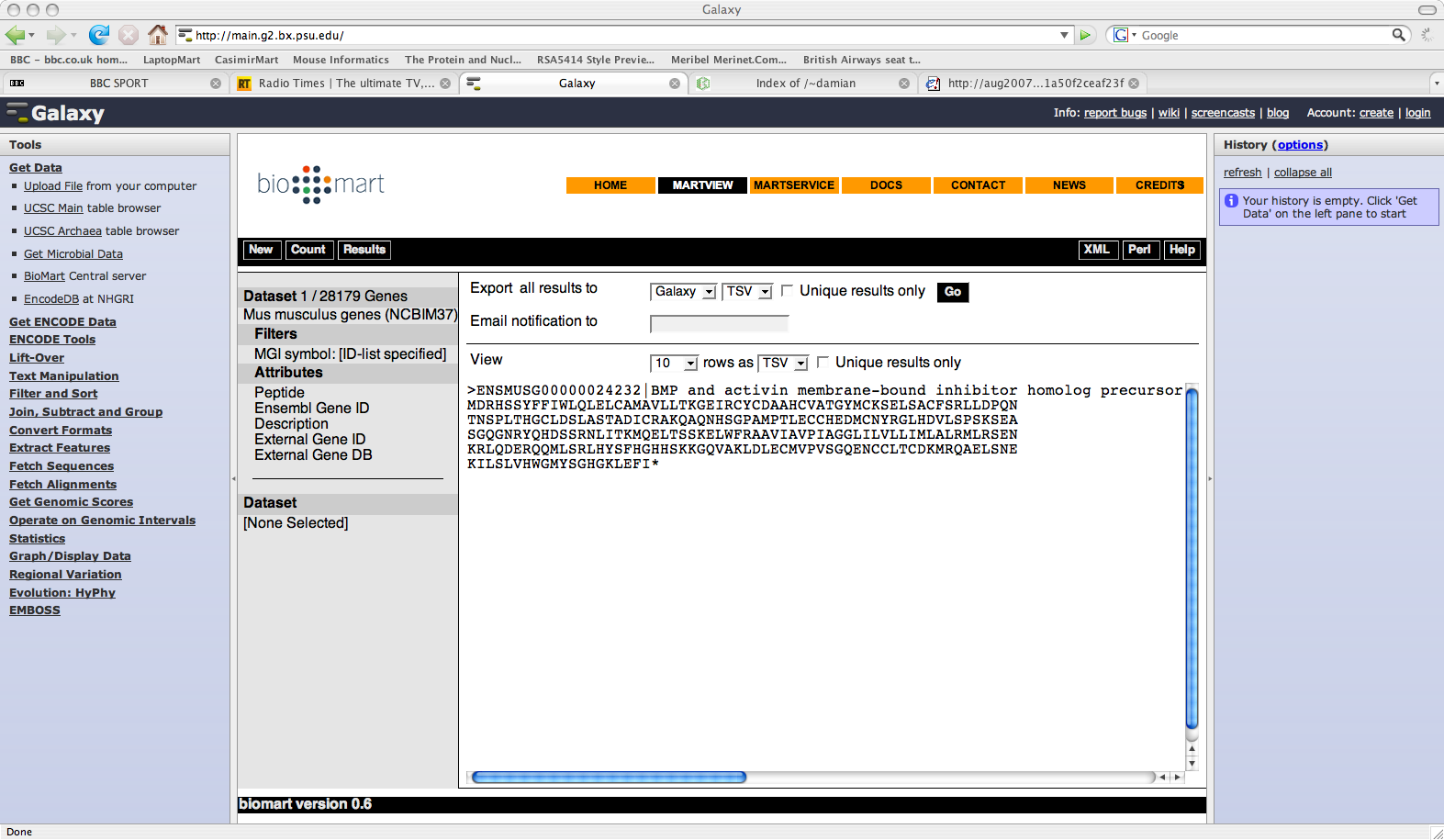 |
| --- | --- |
| **B** | **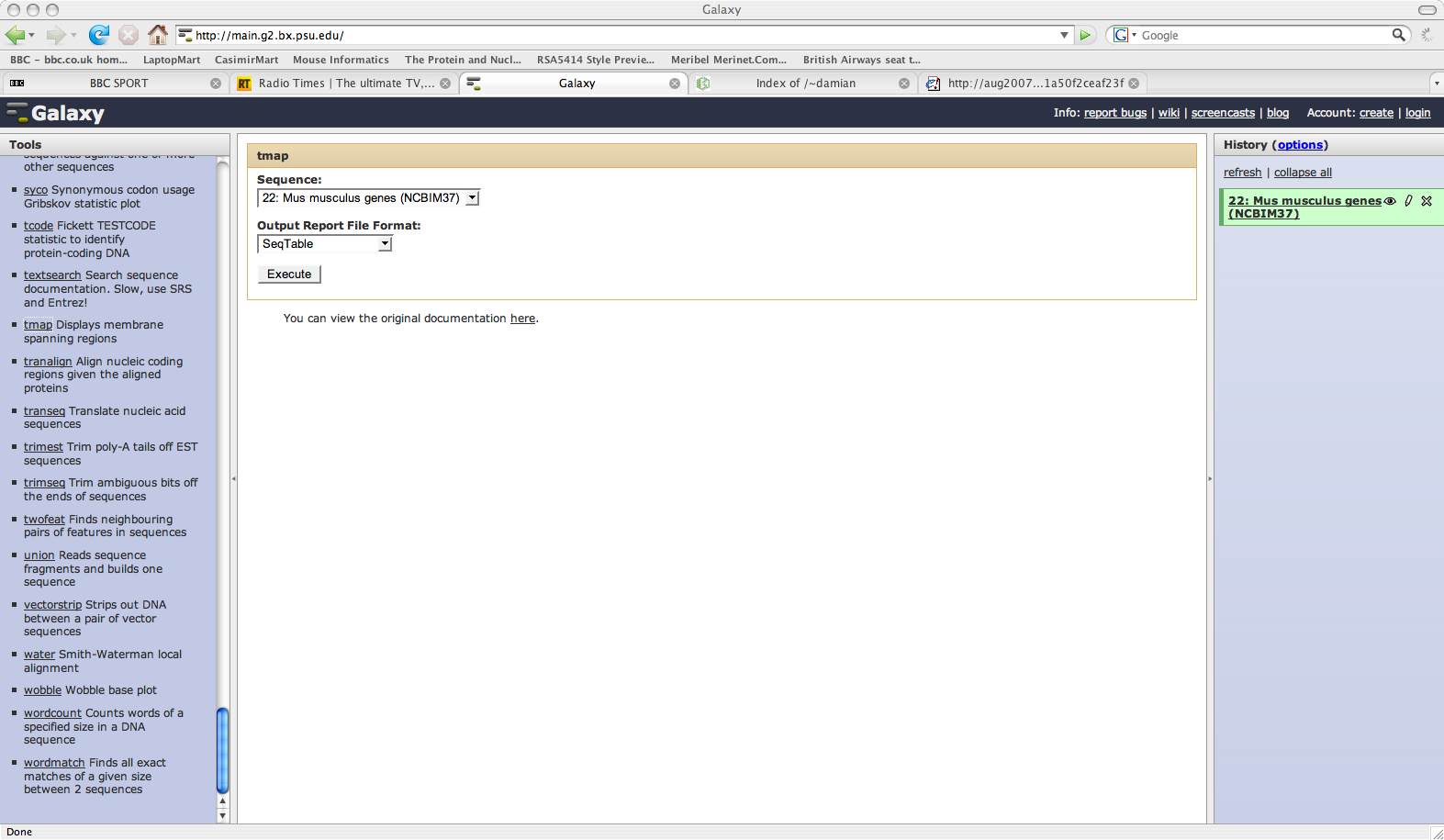** |
| **C** | #=======================================  #  # Sequence: Consensus from: 1 to: 261  # HitCount: 2  #=======================================  Start End TransMem Sequence  8 32 1 FFIWLQLELCAMAVLLTKGEIRCYC  148 176 2 LWFRAAVIAVPIAGGLILVLLIMLALRML  #--------------------------------------- |
