# Supplementary figures and images for: BioMart – biological queries made easy
Source: BMC Genomics. 2009 Jan 14;10:22. doi: 10.1186/1471-2164-10-22 (PMC2649164; doi:10.1186/1471-2164-10-22)

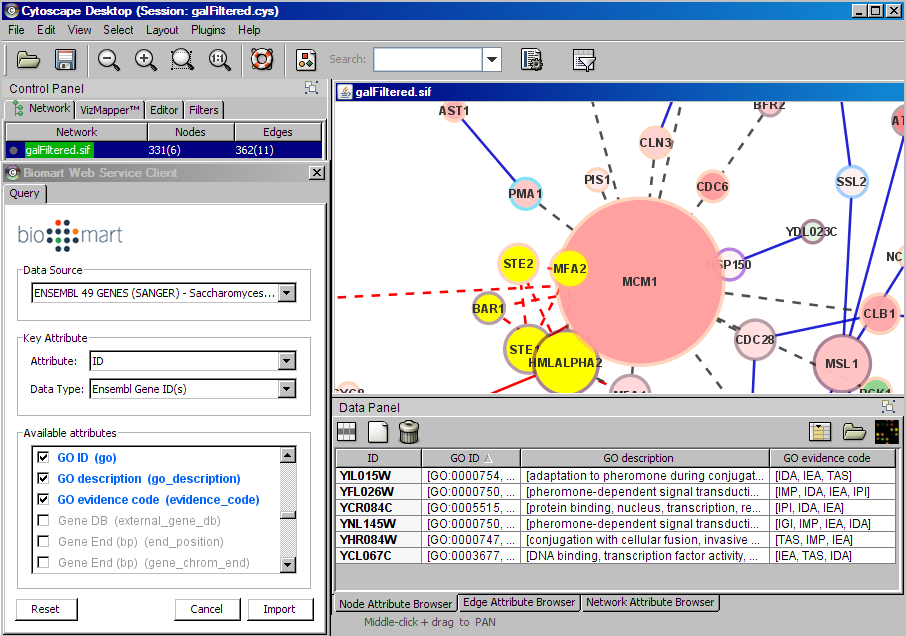

Supplement: Additional file 3 — Cytoscape platform used to visualise a yeast protein interaction network. Annotation of the selected nodes in yellow is shown in the bottom pane and uses MartServices on our central portal to retrieve the GO annotation for each node. [file 1471-2164-10-22-S3.doc]

| 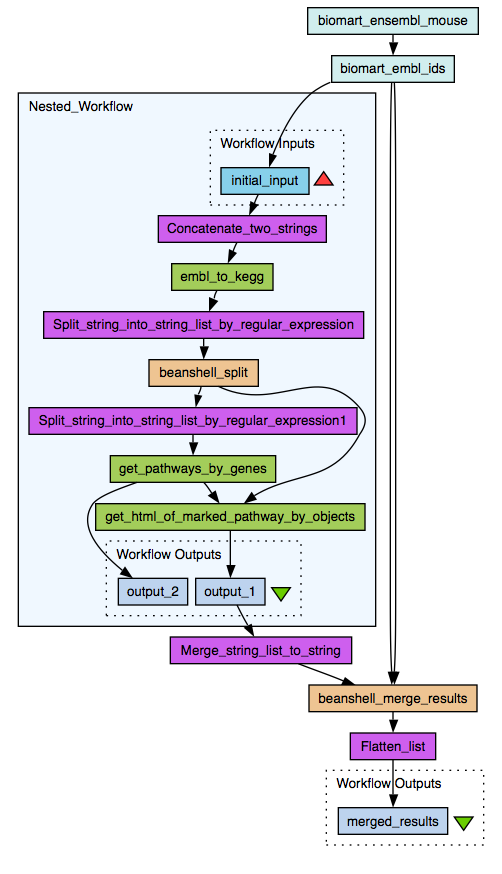 |  | | 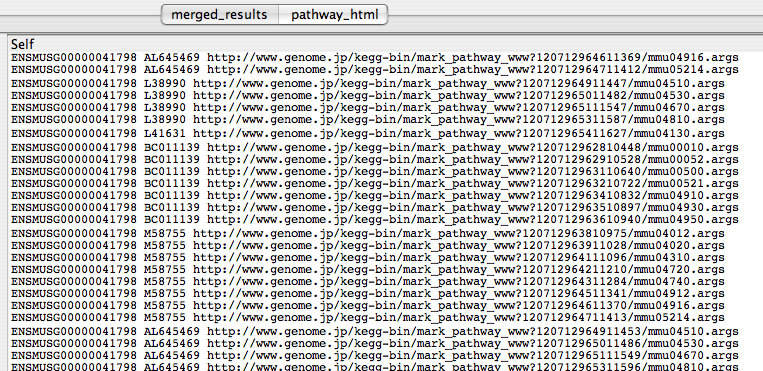 | | --- | | 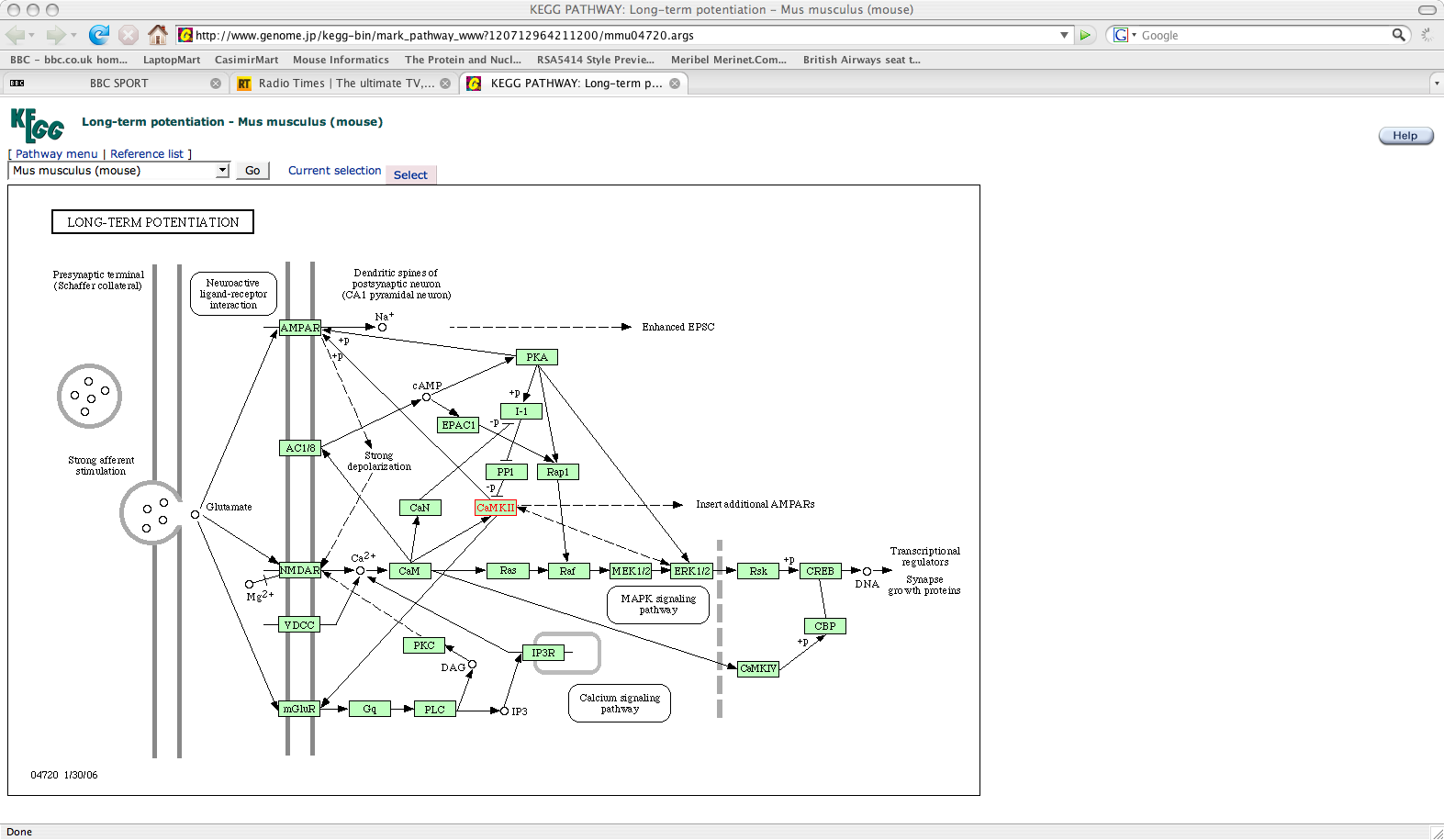 | |
| --- | --- | --- | --- | --- |

Supplement: Additional file 4 — Taverna workflow demonstrating BioMart and web services interaction. Ensembl Gene IDs and EMBL IDs for a given set of genes (results of an Affymetrix microarray experiment) are recovered. The left hand panel shows a graphical depiction of the workflow in which the EMBL IDs are converted to KEGG IDs and then HTML links to marked up pathways using KEGG web services. The upper right panel shows the tabular results of the workflow with Ensembl Gene IDs mapped to KEGG pathway URLs. The bottom right panel shows one of these links with the mapped gene marked in red in the pathway. [file 1471-2164-10-22-S4.doc]

| **A** | **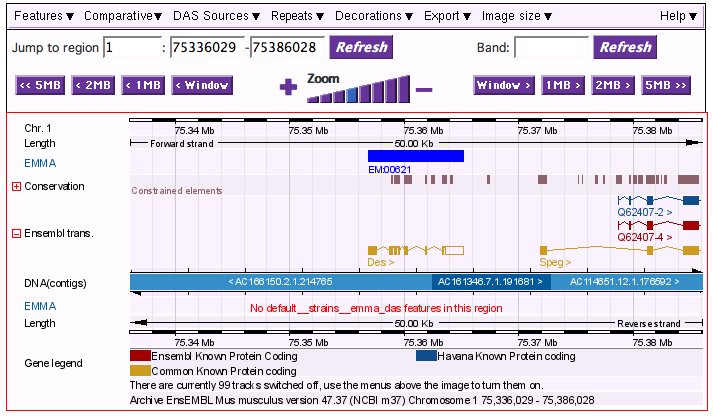** |
| --- | --- |
| **B** | **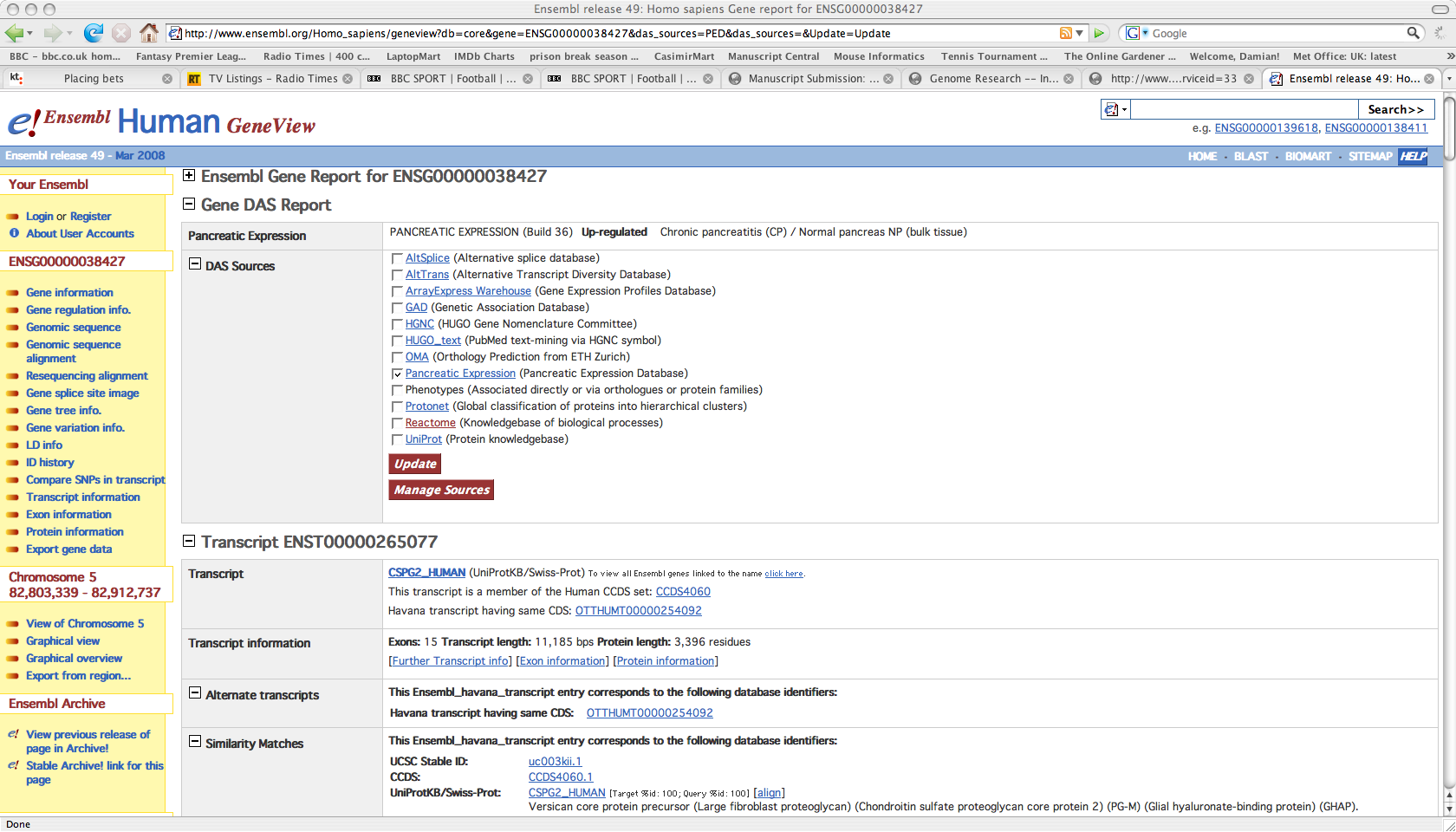** |

Supplement: Additional file 5 — BioMart as a DAS server. Ensembl ContigView display showing a EMMA mouse strain archive track in blue (A). The data is served using the DAS protocol from a BioMart server in an external location to the rest of the Ensembl data. Ensembl GeneView showing Pancreatic Expression Database annotation (B). This annotation comes from a geneDAS source served by the BioMart server. [file 1471-2164-10-22-S5.doc]
